# The Proton-Sensing G-Protein Coupled Receptor GPR4 Promotes Angiogenesis in Head and Neck Cancer

**DOI:** 10.1371/journal.pone.0152789

**Published:** 2016-04-14

**Authors:** Zhibin Jing, Hongbo Xu, Xiaohong Chen, Qi Zhong, Junwei Huang, Yang Zhang, Wei Guo, Zheng Yang, Shuo Ding, Ping Chen, Zhigang Huang

**Affiliations:** Department of Otolaryngology-Head and Neck Surgery, Beijing Tongren Hospital, Capital Medical University, Beijing, China; Medical College of Wisconsin, UNITED STATES

## Abstract

Squamous cell carcinoma of the head and neck (SCCHN) is an aggressive disease with poor survival and is the sixth most common cancer worldwide. Gastroesophageal reflux is a common event in SCCHN patients. GPR4 is a proton-sensing G-protein coupled receptor, which can be activated by acidosis. The objective of this study was to explore the role of GPR4 in acid exposure and tumor angiogenesis in SCCHN. In this study, we confirmed that overexpressing GPR4 in SCCHN cells could increase the expression and secretion of IL6, IL8 and VEGFA at pH 5.9. This effect could be inhibited by SB203580 (a p38 inhibitor). Western blot analysis indicated that phosphorylation of p38 increased in GPR4 infected cells at pH 5.9, which could be inhibited by SB203580. In tube formation assay, HMEC-1 cells were incubated with conditioned medium (CM, pH 5.9, 6.5, 7.4) derived from control and GPR4 infected SCCHN cells. Tube length was significantly increased in HMEC-1 cells incubated with CM from GPR4 infected cells compared with control cells at pH5.9, which indicated the pro-angiogenic effect of GPR4 in acidic pH. The neutralizing antibodies of IL6, IL8 and VEGFA could inhibit tube formation of HMEC-1 cells. In vivo, the effect of GPR4 on angiogenesis was investigated with the chick chorioallantoic membrane (CAM) model. Control and GPR4 infected SCCHN cells were seeded onto the upper CAM surface (n = 5 in each group) and 5 μL DMEM/F12 (pH 5.9, 6.5, 7.4) was added to the surface of the cell every 24 h. Four days later, the upper CAM were harvested and the ratio of the vascular area to the CAM area was quantified using Image-Pro Plus 6.0 software. GPR4 infected cells could recruit more vascular than control cells at pH5.9. In conclusion, we suggested that GPR4 induces angiogenesis via GPR4-induced p38-mediated IL6, IL8 and VEGFA secretion at acidic extracellular pH in SCCHN.

## Introduction

Squamous cell carcinoma of the head and neck (SCCHN) is an aggressive disease with poor survival and is the sixth most common cancer worldwide [1]. The global incidence is approximately 600,000 cases each year [2]. Smoking and alcohol abuse are major risk factors for SCCHN. Gastroesophageal reflux is a common event in SCCHN patients [3], which indicates that it is associated with an elevated risk of laryngeal cancer and pharyngeal cancer [4]. Twenty-four-hour double-probe pH monitoring indicates the pH of the upper esophageal sphincter as being below 4 [5]. Acid exposure can lead to various otolaryngological disorders such as chronic laryngitis, vocal nodules, Reinke’s edema, contact ulcer and granuloma, laryngeal stenosis, and paroxysmal laryngospasm [6]. In esophageal squamous cell carcinoma, continuous acid exposure promotes vascular development [7], which plays a critical role during tumor initiation and malignant progression [8].

The proton-sensing G protein-coupled receptors (GPCRs) including GPR4, GPR65 (TDAG8), GPR68 (OGR1), and GPR132 (G2A) have recently been identified as novel pH sensors that are proposed to be activated by acidic extracellular pH [9]. It has been demonstrated that GPR4 is overexpressed in various types of malignancies [10, 11]. In epithelial ovarian carcinoma, GPR4 expression density is accompanied by a higher microvascular density (MVD) [10], and this correlated with the status of lymph node metastasis and clinical stage. This indicates that GPR4 may be involved in cancer-related angiogenesis. In SCCHN, the correlation between acid exposure, tumor angiogenesis and GPR4 has not been well studied. In a previous study, we confirmed that GPR4 could activate AP1 and ERK signaling pathways at acidic extracellular pH [12]. AP1 is one of the regulatory sites of IL6, IL8 and VEGFA [13–15]. GPR4 as a pH sensor is positively correlated with higher microvascular density, so we hypothesized that it could increase the expression of IL6, IL8 and VEGFA and induce angiogenesis at acidic extracellular pH. In this study, we suggested that GPR4 induced angiogenesis via GPR4-induced IL6, IL8 and VEGFA secretion at acidic extracellular pH in SCCHN.

## Materials and Methods

### Ethics Statement

Ethical permission for this study was granted by the Research Ethics Committee of Beijing Tongren Hospital (Beijing, China).

### Cell Culture

The SCCHN cell lines FaDu cells and Tca8113 cells, human microvascular endothelial cells (HMEC-1) were used in the study. FaDu cells were obtained from State Key Laboratory of Oral Diseases, Sichuan University [16]. Tca8113 cells were obtained from China Infrastructure of Cell Line Resources. HMEC-1 cells, obtained from Thermo Fisher, was generated by Ades et al [17]. All cells were cultured in Dulbecco’s modified Eagle’s medium (DMEM, Hyclone, USA) supplemented with 10% fetal bovine serum (FBS, Gibco) and 1% penicillin/streptomycin. All cells were incubated at 37°C in a humidified atmosphere containing 5% CO_2_.

### Generation of Recombinant Adenovirus Carrying GPR4 Gene

The recombinant adenovirus expressing GPR4 (Ad-GPR4) was generated according to the methods described previously [18]. Briefly, GPR4 was sub-cloned into the adenoviral vector pAdxsi (Sinogenomax, China). The recombinant adenoviral construct was transfected into the packaging HEK293 cells and then these cells were freeze–thawed several times to release intracellular viral particles. The viral titers were tested in HEK293 cells and the number of plaque-forming units (pfu) was obtained using a plaque formation assay.

### Cell Infection and Acid Stimulation

FaDu cells and Tca8113 cells were seeded in six-well plates, reaching 80% confluence by the following day. Then the cells were infected with Ad-GPR4 or Ad-null (a blank recombinant adenovirus as control). Eighteen hours later, acid stimulation was performed as described previously [12]. Briefly, 18h after infection, the culture media were replaced by serum-free DMEM/F12 (pH 5.9, 6.5 and 7.4). The cells were harvested after stimulation for 6h. SB203580 (a p38 inhibitor, 1 μM) was used to determine whether the p38 mediated the secretion of the pro-angiogenic cytokines. To perform p38 inhibition, GPR4 infected cells were serum-starved for 4h and treated with 1 μM SB203580 for 1h before acid stimulation and then the culture media were replaced by serum-free DMEM/F12 (pH 5.9). The cells were harvested 6h later.

### Conditioned Medium

To prepare conditioned medium (CM), the FaDu cells and Tca8113 cells were infected with Ad-GPR4 or Ad-null for 18h, the culture media were replaced by serum-free DMEM/F12 (pH 5.9, 6.5 and 7.4), and cells without infection acted as a control. Six hours later, the medium was centrifuged (4°C, 1500 rpm) and the supernatant was collected and stored at –80°C until use.

### Real Time PCR (qPCR) and Semiquantitative PCR

Total RNAs of FaDu and Tca8113 cells and SCCHN tissues were isolated using TRIzol Reagent (Life Technologies, USA). Reverse transcription was performed using a FastQuant RT Kit (TIANGEN, China). qPCR was performed as recommended by the manufacturer. The following primers were used for qPCR: IL6 sense 5′-TGAGAGTAGTGAGGAACAAG-3′, antisense 5′-CGCAGAATGAGATGAGTTG-3′; IL8 sense 5′-CCTGATTTCTGCAGCTCTGTGT-3′, antisense 5′-GGTGGAAAGGTTTGGAGTATGTCT-3′; VEGFA sense 5′-GCCCACTGAGGAGTCCAACATC-3′, antisense 5′-CAAGGCCCACAGGGATTTTCT-3′. The primers for PCR of GPR4 were as follows: sense 5′-AACCTCTATCGGGTGTTCGTG-3′, antisense 5′-TTGGCTGTGCTGTTCCTCTTGG-3′. GAPDH was amplified as an internal standard. The relative RNA level in each sample was determined by the 2(–Delta Delta C(T)) method.

### Enzyme-Linked Immunosorbent Assay (ELISA)

The control cells and GPR4 infected cells were stimulated for 6h using serum-free DMEM/F12 (pH 5.9). Then the medium was centrifuged (4°C, 1500 rpm) and the supernatant was collected. The concentration of IL6, IL8, and VEGFA in the culture medium was quantified with an ELISA kit according to the manufacturer’s instructions (Abcam).

### Western Blot Analysis

Cells were lysed in RIPA buffer (Beyotime, China) supplemented with a 1 mM PMSF. A total of 100 μg proteins were electrophoresed on 10% SDS–PAGE gels, and electrotransferred onto nitrocellulose membranes (Invitrogen, USA). Western blot was performed as previously described [12]. GPR4 antibody was purchased from LifeSpan BioSciences (LifeSpan, USA). Phospho-p38 (p-p38), total p38 (p38), phospho-VEGF receptor 2 (Tyr1175) and VEGF receptor 2 antibody were purchased from Cell Signaling Technology (Cell Signaling, USA). GAPDH (Santa Cruz, CA) was used as a lysate loading control.

### Tube Formation Assay

To confirm the effect of GPR4 on tube formation in HMEC-1, we performed an angiogenesis assay on growth factor-reduced Matrigel (BD Biosciences). One day before tube formation assay, HMEC-1 cells were seeded on a 10-cm disk. After coating the 96-well plate with Matrigel following the manufacturer’s instructions, HMEC-1 cells were harvested and resuspended at 1×10^5^/mL using CM supplemented with 2% FBS. Then 0.1 mL of cell suspension was added to the 96-well plate. VEGFA (1 ng/mL) was used as a positive control and DMEM served as a negative control. The plate was incubated at 37°C, 5% CO_2_ for 12h to allow formation of capillary-like structures. Pictures were taken using a light microscope and the tube length (pixels) was counted using Image-Pro Plus 6.0 software.

### Chick Chorioallantoic Membrane (CAM) Model

Fertilized Specific Pathogen Free (SPF) eggs were obtained from Beijing Laboratory Animal Research Center. Eggs were hatched in a humidified incubator at 37.0°C with 60% humidity. On day 10, large blood vessels and sac were labeled on the egg using Cold Light Fountain (STORZ, Germany). After disinfection using 75% alcohol, a round window was opened on the top of the eggshell, maintaining the outer eggshell membrane. A pinpoint hole was drilled at the location of the air sac. Then 20 μL of normal saline was added to the eggshell membrane. The eggshell membrane was punctured using a fine needle and the small pinpoint hole in the air sac was vacuumed using a rubber pipette bulb, so that the normal saline separated the outer eggshell membrane from the CAM. The eggshell membrane was peeled off without disturbing the CAM. The window was covered with parafilm and the egg was placed in the incubator for another day. Then 1×10^6^control and GPR4 infected cells were seeded onto the upper CAM surface (n = 5 in each group). The square window on the egg was sealed. Then 5 μL DMEM/F12 (pH 5.9, 6.5, 7.4) was added to the surface of the cell every 24h. Four days later, the upper CAM were harvested and the ratio of the vascular area to the CAM area was quantified using Image-Pro Plus 6.0 software.

### Bioinformatics

Bioinformatics method was used as Li [19] described, briefly, array data related to cancers from the Affymetrixhuman genome U133 plus 2.0 platform were down loaded from the GEO database (http://www.ncbi.nlm.nih.gov/geo/), and differentially expressed genes in tumors were analyzed using TumourProfile database (http://tumour.bjmu.edu.cn/, unpublished) as Wang et al previously described data processing and microarray analysis methods [20, 21]. The expression profile of GPR4 in a variety of cancers and the corresponding control (normal or non-tumor) tissues was searched in this database, and the expression levels were represented as average rank scores (ARS).

### Immunohistochemistry (IHC)

Paraffin-embedded specimens of laryngeal or hypopharyngeal squamous cell carcinoma samples coupled with peri-carcinoma normal tissue samples were obtained from the head and neck tumor specimen bank of Beijing Tongren Hospital [12]. Paraffin tissue slides were dewaxed, rehydrated and placed in 10 mmol/L citrate buffer (pH 6.0), and heated twice in a microwave oven for 5 min each. Slides were incubated with 3% H2O2 for 10 min, washed with PBS, blocked with 10% normal goat serum for 30 min, and then incubated with anti-GPR4 (diluted 1:100; final concentration, 2 μg/mL, LifeSpan, USA) or normal rabbit IgG as a control at 4°C overnight. After washing, the slides were stained with the catalyzed signed amplification system kit (DAKO code k5007) and images were photographed with an Olympus IX70 microscope. Semiquantitative expression of GPR4 was calculated using Image-Pro Plus 6.0 software.

### Data Analysis

The bioinformatics analysis of the differences in GPR4 expression between the cancers and control tissues were evaluated using the Wilcoxon rank-sumtest in the R (http://www.r-project.org/) software environment. Bonferroni’s correction of the R function “p.adjust” was used to adjust the P-values. The experimental data were analyzed using Student’s t test with SPSS19.0 software. A p-value <0.05 was considered to be statistically significant.

## Results

### GPR4 Stimulates Pro-Angiogenic Cytokine Secretion at Acidic Extracellular pH

**Fig 1:**
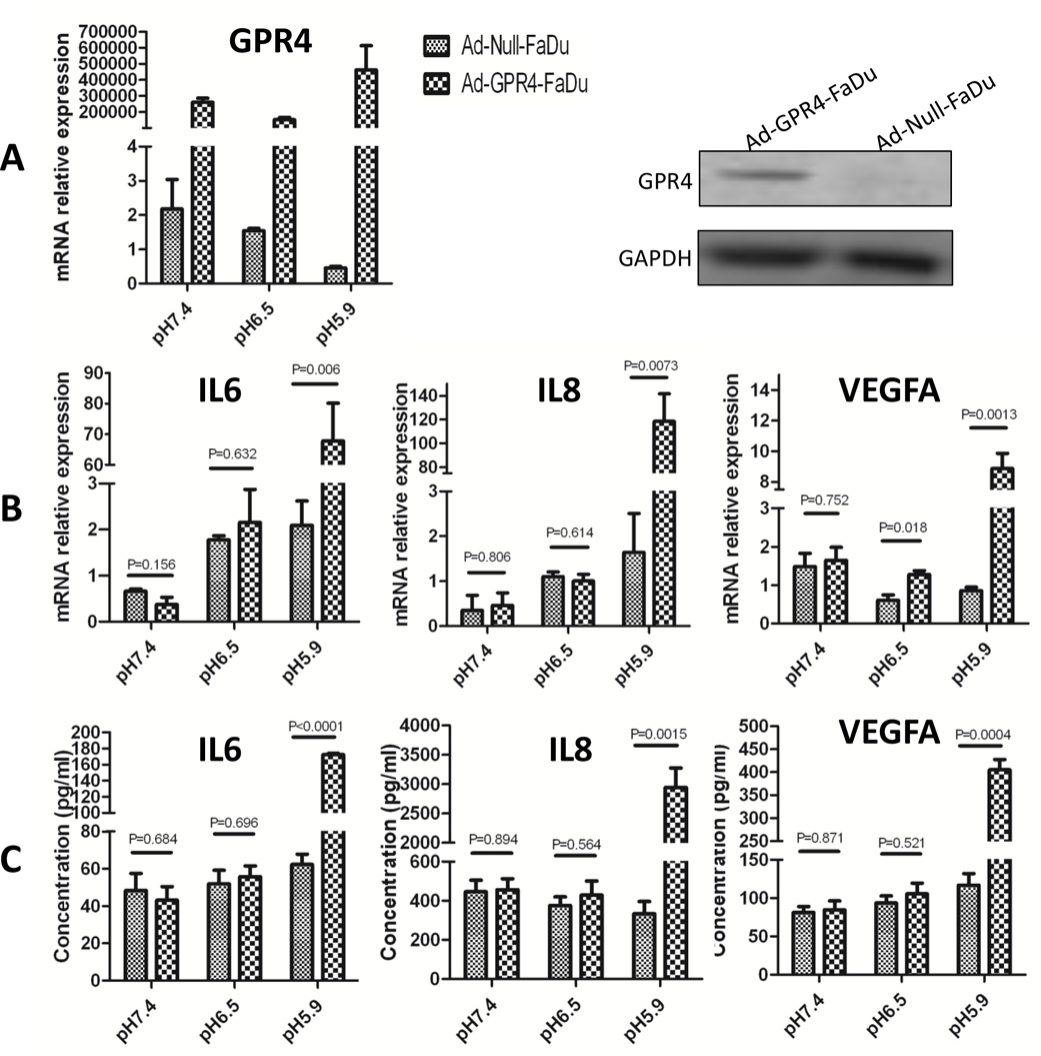
GPR4 stimulates cytokine secretion at acidic pH. Total RNA and protein of Ad-GPR4-FaDu cells, Ad-null-FaDu cells were isolated after acid stimulation for 6 h. qPCR and western blot were performed. **(A)** Overexpression of GPR4 in Ad-GPR4-FaDu cells was comfirmed by qPCR and western blot. **(B)** The expression of IL6, IL8, and VEGFA increased significantly in GPR4 infected cells at pH 5.9. **(C)** The supernatants of the cells were collected and the concentrations of IL6, IL8 and VEGFA were detected by ELISA. Similar results were observed in Tca8113 cells (S1 Fig).

**Fig 2:**
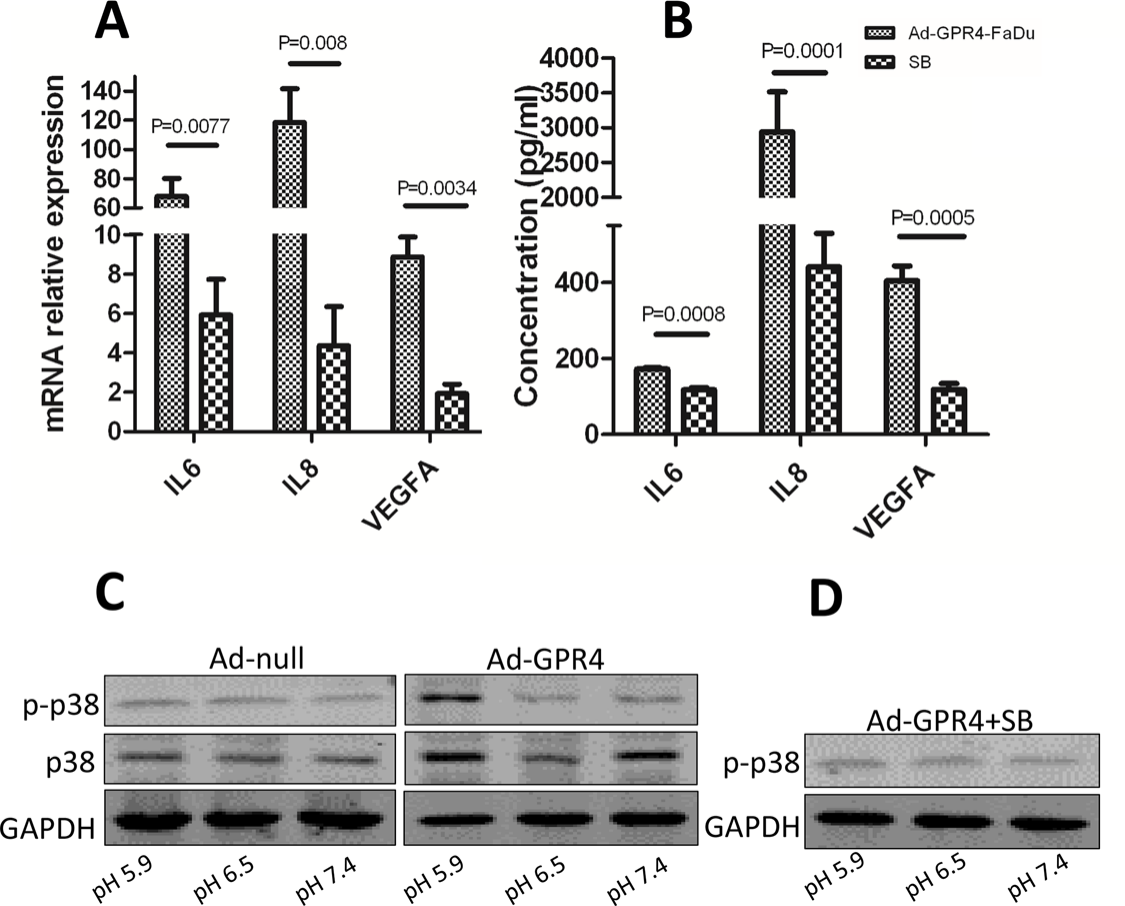
SB203580 reduces cytokine secretion by inhibiting p38 phosphorylation. **(A)** In Ad-GPR4-FaDu cells, SB203580 reduced the expression of IL6, IL8 and VEGFA at pH 5.9. **(B)** SB203580 reduced secretion of IL6, IL8 and VEGFA. **(C)** GPR4 increased p38 phosphorylation at pH5.9. **(D)** SB203580 inhibit phosphorylation of p38 in GPR4 overexpressed cells. Similar results were observed in Tca8113 cells (S2 Fig).

To investigate whether GPR4 could induce the expression of pro-angiogenic cytokines at acidic extracellular pH, we detected the expression of IL6, IL8, and VEGFA at different pH values (pH 7.4, 6.5 and 5.9). The overexpression of GPR4 in GPR4 infected cells was validated by qPCR and western blot (Fig 1A). The mRNA levels of IL6, IL8, and VEGFA in the Ad-GPR4 cells increased statistically significantly compared with the Ad-null cells only at pH 5.9 (Fig 1B). Compared with the controls, there was 67.8-, 118- and 12.5-fold induction for IL6, IL8 and VEGFA, respectively, in the Ad-GPR4 cells. This indicated that acidic extracellular pH enhanced pro-angiogenic cytokine expression via GPR4, which was further confirmed at the protein level. The concentrations of IL6, IL8, and VEGFA were increased significantly in the supernatant of GPR4 overexpressed cells compared with the control cells at pH 5.9 (Fig 1C). The inhibitor, SB203580, could reduce the expression and secretion of IL6, IL8, and VEGFA (Fig 2A and 2B), suggesting that p38 may be involved in cytokine induction by GPR4. Western blot was performed to investigate if GPR4 could increase p38 phosphorylation at different pH. Phosphorylation of p38 increased in GPR4 overexpressed cells at pH 5.9 (Fig 2C), which could be inhibited by SB203580 (Fig 2D). Our results was correspondent with that, in SCCHN, p38 is constitutively activated and leads to increasing secretion of cytokines IL6 and IL8 [22].

### GPR4 Promotes Angiogenesis In Vitro

**Fig 3:**
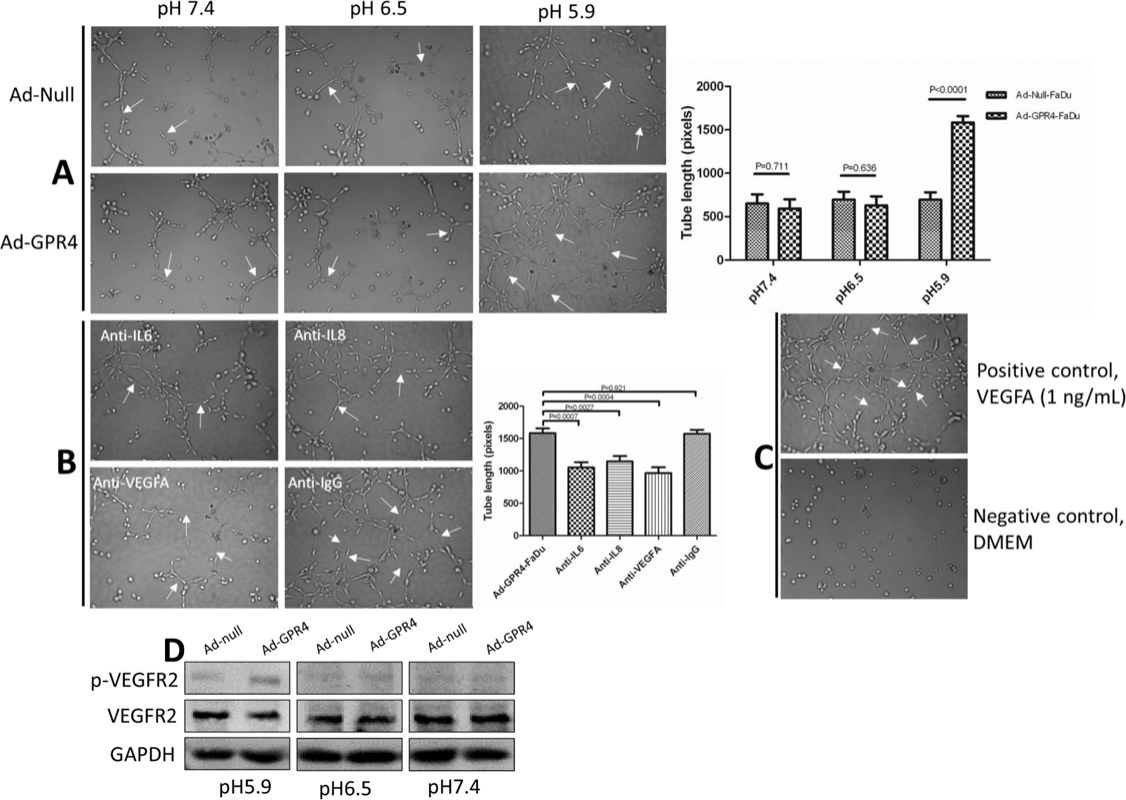
Tube formation assay. **(A)** The tube length (arrows) of HMEC-1 cells was increased in CM derived from Ad-GPR4-FaDu cells compared with Ad-null-FaDu cells at pH 5.9. **(B)** The neutralizing antibodies of IL6, IL8 and VEGFA inhibited tube formation in HMEC-1 cells. Isotype IgG antibody was used as a control in neutralizing antibody test. **(C)** VEGFA (1 ng/mL) was used as a positive control and DMEM served as a negative control in tube formation assay. Tube lengths (pixels) were quantified from 6 representative fields using Image-Pro Plus 6.0 software. **(D)** GPR4 increased VEGFR2 phosphorylation in HMEC-1 cells at pH 5.9. Similar results were observed in Tca8113 cells (S4 Fig).

To test whether GPR4 could promote tube formation, HMEC-1 cells were incubated with CM derived from control cells and GPR4 overexpressed cells. The lengths of tubes (arrows) were significantly increased in HMEC-1 cells incubated with CM from GPR4 infected cells compared with control cells at pH 5.9 (Fig 3A). This indicated that overexpression of GPR4 in SCCHN could induce angiogenesis in vitro at acidic pH. In SCCHN, angiogenesis is triggered by IL-8, IL-6 and VEGF [23, 24]. To test whether the effect of GPR4-induced angiogenesis was directly mediated by IL6, IL8 and VEGFA secretion, monoclonal neutralizing antibodies (R&D Systems) of IL6, IL8 and VEGFA were added to the CM of Ad-GPR4-FaDu cells, respectively, isotype IgG antibody was used as a negative control (Fig 3B). These neutralizing antibodies reduced the tube length of HMEC-1, and moreover, the neutralizing antibody effects could be reversed by the addition of the excess amount of these factors (S3 Fig). This suggested that GPR4 induced angiogenesis via IL6, IL8 and VEGFA secretion. Vascular endothelial growth factor receptor 2 (VEGFR2) is the primary receptor of VEGF and the major mediator of VEGF-induced pro-angiogenesis signaling in endothelial cells. To test whether GPR4 increased VEGFR2 phosphorylation in HMEC-1 cells, western blot was performed. The results showed that VEGFR2 phosphorylation increasing in HMEC-1 cells incubated with CM (pH 5.9) derived from GPR4 overexpressed cells (Fig 3D).

### GPR4 Promotes Angiogenesis In Vivo

**Fig 4:**
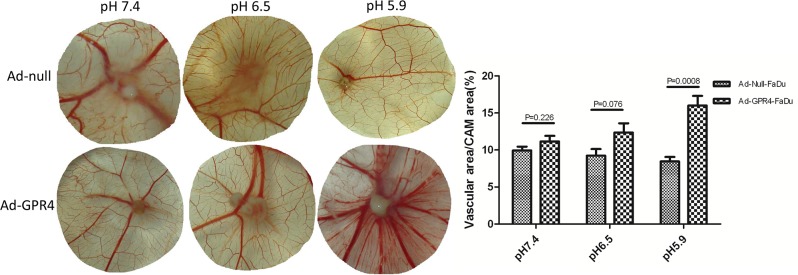
GPR4 induced angiogenesis in CAM model. The Ad-GPR4-FaDu cells recruited more vascular than Ad-null-FaDu cells only at pH 5.9. No differences were observed between Ad-GPR4-FaDu cells and Ad-GPR4-FaDu cells at pH 7.4 and 6.5. The ratios of vascular area to CAM area were quantified using Image-Pro Plus 6.0 software. Similar results were observed in Tca8113 cells (S5 Fig).

In vivo, the angiogenic potential of GPR4 in SCCHN was verified in the CAM model. GPR4 infected cells seeded on the CAM recruited more vascular than control cells at pH 5.9. The vascular density was quantified by calculating the ratio of the vascular area to the CAM area. In the Ad-null and Ad-GPR4-FaDu cells, the vascular densities surrounding the tumor were 8.46%, and 16%, respectively (Fig 4). This indicated that overexpression of GPR4 in SCCHN could promote angiogenesis in vivo at acidic pH. The in-vivo and in-vitro studies indicated that GPR4 induces angiogenesis at acidic pH, a hallmark of tumor progression.

### GPR4 Is Overexpressed in SCCHN

GPR4 is overexpressed in various types of malignancies including breast cancers, ovarian cancers, colon cancers, liver cancers and kidney cancers [11]. To assess the differential expression of GPR4 across multiple cancers and their corresponding control (normal or non-tumor) tissues at the mRNA level, an integrated bioinformatics analysis were performed based on the omic tumor data set from the GEO database. The average rank scores (ARS), Bonferroni correction adjusted P-values (Table 1) were synthesized, and GPR4 was upregulated in kidney clear cell renal cell carcinoma, SCCHN and colorectal adenocarcinoma. The overexpression of GPR4 in SCCHN is further supported by the BioXpress (http://hive.biochemistry.gwu.edu/tools/bioxpress) [25]. The BioXpress database indicates that GPR4 is upregulated in 85.37% of SCCHN samples compared with their paired normal samples based on RNA sequencing (RNA-seq); the data set deposited in the Cancer Genome Atlas (TCGA) from a total of 72 patients has been collected and used for the analysis [25].

**Table 1:** The average expression intensities of GPR4 in multiple cancers.

Tissue	Sample size	ARS^a^	P value^b^	Bonferroni^c^
bladder cancer	186	56.15	0.780492675	1
bladder control	64	55.34		
breast cancer	3080	55.68	0.019528491	1
breast control	374	55.94		
colorectal adenocarcinoma^d^	1841	55.36	9.56E-25	5.21E-20
colon mucosa normal	273	47.41		
gastric cancer	681	58.35	0.132038546	1
gastric normal	61	56.64		
SCCHN^d^	220	63.44	7.27E-06	0.397103264
SCCHN control	59	51.29		
kidney clear cell renal cell carcinoma^d^	652	74.97	2.77E-77	1.51E-72
kidney control	244	56.72		
hepatocellular carcinoma	263	53.96	0.11868146	1
liver control	62	56.89		
oral squamous cell carcinoma	339	53.82	4.97E-06	0.271182795
oral squamous cell control	118	48.25		
ovarian cancer	379	54.19	0.013964164	1
ovary control	120	50.28		
pancreatic cancer	263	57.04	0.166179535	1
pancreas control	83	59.98		
prostate cancer	345	58.62	0.46249681	1
prostate control	81	59.41		

^a^ARS denotes the average rank score.

^b^The P-values were calculated using the Wilcoxon rank-sum test in the R (http://www.r-project.org/) software environment and are relative to the corresponding normal or non-tumor tissues.

^c^The P-values were adjusted using Bonferroni correction in the function “p.adjust” in the R software.

^d^The expression levels of GPR4 in the underlined tissues were considered to be upregulated by fully considering the differences in the ARS values, the Bonferroni correction adjusted P-values.

**Fig 5:**
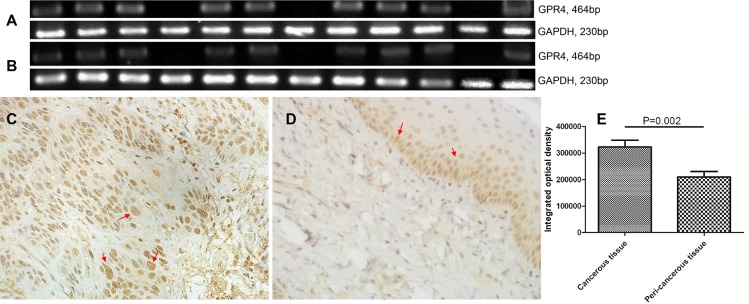
GPR4 is overexpressed in SCCHN. **(A)** RT-PCR of GPR4 in cancerous tissues. **(B)** RT-PCR of GPR4 in peri-cancerous normal tissues. GAPDH was used as an internal standard. **(C)** Representative image of IHC of moderately differentiated hypopharyngeal cancer. **(D)** Peri-cancerous normal tissue from the same patient as C. **(E)** Integrated optical density (IOD) of positive expression cells (arrows) was calculated using Image-Pro Plus 6.0 software. In total, 12 cases of SCCHN were included. The level of immunoreactivity was graded by calculating the integrated optical density (IOD) of positive expression cells.

We collected 12 SCCHN samples (Table 2) and performed RT-PCR (Fig 5A and 5B) and immunohistochemical (IHC) staining (Fig 5C and 5D). All of the patients suffered from gastroesophageal reflux. As shown in Fig 5, GPR4 was amplified in 9/12 samples and upregulated in the tumor tissues (Fig 5A) compared with the peri-tumor normal tissues (Fig 5B). The GPR4 protein expressed at higher levels (red arrows) in the tumor cells compared with the epithelial cells of peri-tumor normal tissues. Similar results were observed in both laryngeal and hypopharyngeal cancers.

**Table 2:** Clinical features of the SCCHN samples and corresponding GPR4 expression in IHC.

Case	Gender	Age	Clinical and pathological diagnosis	TNM staging	Differentiation	IOD of CT	IOD of PCT
1	M	59	SGC	T2N0M0	High	352567.565	255943.18
2	M	74	GC	T2N0M0	Moderate	396264.045	173284.705
3	M	67	GC	T3N2M0	High	249689.9	197848.23
4	M	47	GC	T4N0M0	Moderate	340095.92	307834.09
5	M	78	GC	T3N0M0	Moderate	379242.5	279154.405
6	M	66	HPC	T2N2M0	Moderate	489210.845	219239.21
7	M	57	HPC	T3N1M0	Moderate	275309.335	245821.32
8	M	50	HPC	T3N2M0	Low	379145.465	134423.65
9	M	69	HPC	T3N1M0	Low	306495.335	153739.275
10	M	71	HPC	T2N1M0	Moderate	313586.58	350465.55
11	M	61	HPC	T2N1M0	Low	273720.055	109265.1685
12	M	56	HPC	T3N2M0	Moderate	357452.75	222803.135

CT: cancer tissue; PCT: peri-cancer tissue; IOD: integrated optical density; SGC: supraglottic cancer; GC: glottic cancer; HPC: hypopharyngeal cancer; All cases were squamous cell carcinoma. TNM staging is according to the AJCC cancer staging, 7th edition.

## Discussion

In this study, we have suggested that, when exposed to an acidic environment, GPR4, one of the proton-sensing GPCRs, could induce angiogenesis via pro-angiogenic cytokine secretion in SCCHN. The cytokines included IL6, IL8, and VEGFA, and this was confirmed by qPCR and ELISA. The pro-angiogenic effect of GPR4 was observed by the tube formation assay and CAM model.

Angiogenesis is one of the hallmarks of cancer [26]. Tumors cannot grow beyond a critical size or metastasize to another organ without blood vessels [27], so they must recruit new blood vessels by angiogenesis. The ‘angiogenic switch’ is balanced by pro-angiogenic and anti-angiogenic molecules [27]. One of the signals that trigger this switch is low pH. In human melanoma cells, acidic pH increases the expression of the proangiogenic factors VEGFA and IL8 [28]. GPR4 predominantly functions as a proton sensor and is expressed endogenous in HMEC-1 and HUVEC. Extracellular acidosis can activate GPR4 and lead to cAMP production [29]. Hypercapnic acidosis can also activate GPR4 to stimulate HUVEC adhesion molecule expression and adhesiveness [30]. In another study, it was demonstrated that GPR4 played pivotal roles in growth, migration, and tube formation by endothelial cell, but pH changes could not regulate cAMP production in HMEC-1 and HUVEC [31]. In HUVEC, acidosis activates GPR4 and increases the expression of IL1, IL2, IL6 and IL8 [32]. GPR4, as a pH sensor, is fully activated at acidic pH 6.8 and partially activated in the physiological range (pH 7.4–6.8) [33, 34]. Gastroesophageal reflux is a common event in head and neck cancer patients [3], which means that mucosa and carcinoma are often exposed to low pH. The relationship between low pH and tumor angiogenesis in SCCHN has not been well studied. In this study, we suggested that, in Ad-null-FaDu and Ad-null-Tca8113 cells, low pH could not increase the expression of IL6, IL8, and VEGFA. In Ad-GPR4 cells, the expression of IL6, IL8, and VEGFA increased significantly compared with control at pH 5.9 but not at pH 6.8 and pH 7.4, which may indicate that GPR4 is fully activated at pH 5.9 in SCCHN and this is consistent with our previous study [12].

The tube formation assay and CAM model verified the pro-angiogenic effect of GPR4. CM derived from Ad-GPR4-FaDu cells induced longer tubes in HMEC-1 cells and more vascular density in CAM than in the other groups at pH 5.9. The neutralizing antibodies of IL6, IL8 and VEGFA reduced the tube lengths of HMEC-1 cells, which indicates that GPR4 induced angiogenesis via IL6, IL8 and VEGF secretion. VEGFA and IL6 are recognized as the main pro-angiogenic molecules in SCCHN and have been linked with disease aggressiveness and poor patient outcome [35–40]. IL8, an end-product of the VEGF pathway, was also observed at high levels in SCCHN [40]. Because of the important role of angiogenesis in tumor progression, antiangiogenic agents have been developed. In FaDu SCCHN xenografts, long-term administration of bevacizumab, a vascular endothelial growth factor antibody, effectively modulated chemotherapeutic efficacy [41]. In phase II clinical trials in SCCHN patients, bevacizumab plus cetuximab or chemotherapy showed encouraging efficacy results [42, 43]. Overexpression of IL8 led to resistance to bevacizumab in SCCHN, so co-targeting of VEGF and IL8 may help overcome resistance and enhance therapeutic efficacy.

Antacid medications including histamine 2 receptor antagonist (H2RA) and proton pump inhibitors (PPI) are always used in the treatment of SCCHN patients. Some studies have revealed that the usage of antacid medications may have significant therapeutic benefits for SCCHN patients [44, 45]. One of the mechanisms may be because antacid medications can modulate adhesion of tumor cells to endothelium [44]. In the present study, we have shown that GPR4 promotes pro-angiogenic factors secretion at acidic pH. These results indicate that inhibition of GPR4 or acidic pH may lead to inhibition of tumor angiogenesis. So we speculate that another probable mechanism is that antacid medications can inhibit pro-angiogenic factors secretion by increasing the pH, and then reduce angiogenesis, which plays a critical role during tumor progression. Previously, we showed that acidic extracellular pH enhances matrix metalloproteinase (MMP) expression via the ERK signaling pathway in GPR4 overexpressed cells. In this study, we showed that GPR4 could increase phosphorylation of p38 at pH 5.9, which could be inhibited by SB203580. SB203580 could reduce the expression of pro-angiogenic cytokines, which may indicate p38 play an important role in mediating the expression of cytokines.

In conclusion, we suggested that GPR4 induces angiogenesis via GPR4-induced p38-mediated IL6, IL8 and VEGFA secretion at acidic extracellular pH in SCCHN.

## Supporting Information

S1 Fig GPR4 stimulates cytokine secretion at acidic pH in Tca8113 cells. Total RNA and protein of Ad-GPR4-Tca8113 cells, Ad-null-Tca8113 cells were isolated after acid stimulation for 6 h. qPCR and western blot were performed. (A) Overexpression of GPR4 in Ad-GPR4-Tca8113 cells was comfirmed by qPCR and western blot. (B) The expression of IL6, IL8, and VEGFA increased significantly in GPR4 infected cells at pH 5.9. (C) The supernatants of the cells were collected and the concentrations of IL6, IL8 and VEGFA were detected by ELISA.
(TIF)

S2 Fig SB203580 reduces cytokine secretion by inhibiting p38 phosphorylation in Tca8113 cells. **(A)** In Ad-GPR4-Tca8113 cells, SB203580 reduced the expression of IL6, IL8 and VEGFA at pH 5.9. **(B)** SB203580 reduced secretion of IL6, IL8 and VEGFA. **(C)** GPR4 increased p38 phosphorylation at pH5.9. **(D)** SB203580 inhibit phosphorylation of p38 in GPR4 infected cells.
(TIF)

S3 Fig The neutralizing antibody effects could be reversed by the addition of the excess amount of IL6, IL8 or VEGFA in tube formation assay. (TIF)

S4 Fig Tube formation assay of Tca8113 cells. (A) The tube length (arrows) of HMEC-1 cells was increased in CM derived from Ad-GPR4-Tca8113 cells compared with Ad-null- Tca8113 cells at pH 5.9. (B) The neutralizing antibodies of IL6, IL8 and VEGFA inhibited tube formation in HMEC-1 cells. Isotype IgG antibody was used as a control in neutralizing antibody test.
(TIF)

S5 Fig The Ad-GPR4-Tca8113 cells recruited more vascular than Ad-null-Tca8113 cells only at pH 5.9 in CAM model. (TIF)

S1 File Excel file containing supporting information data of this study. (XLSX)
