# Supplementary figures and images for: The Proton-Sensing G-Protein Coupled Receptor GPR4 Promotes Angiogenesis in Head and Neck Cancer
Source: PLoS One. 2016 Apr 14;11(4):e0152789. doi: 10.1371/journal.pone.0152789 (PMC4831743; doi:10.1371/journal.pone.0152789)

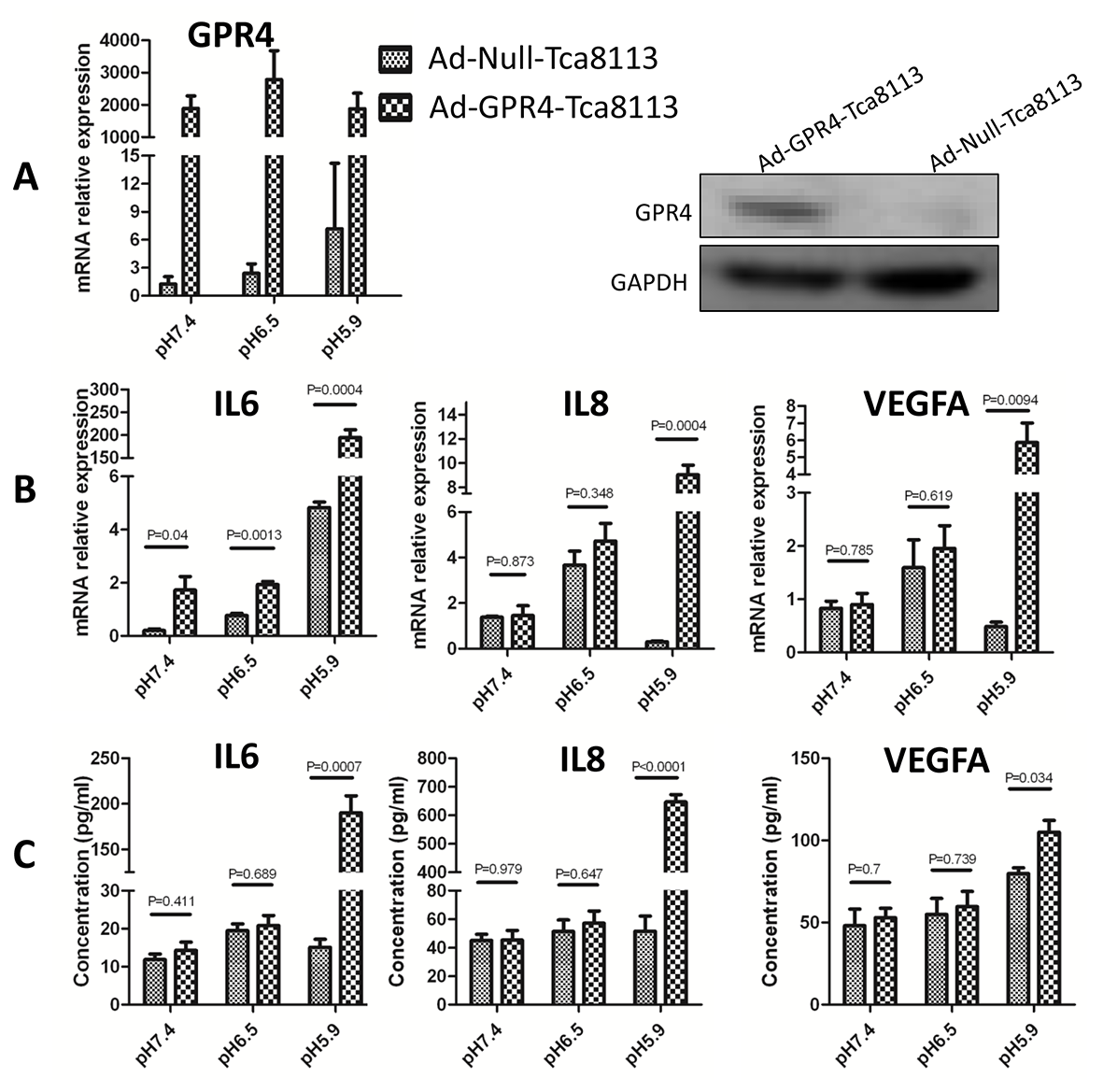

Supplement: S1 Fig — Total RNA and protein of Ad-GPR4-Tca8113 cells, Ad-null-Tca8113 cells were isolated after acid stimulation for 6 h. qPCR and western blot were performed. (A) Overexpression of GPR4 in Ad-GPR4-Tca8113 cells was comfirmed by qPCR and western blot. (B) The expression of IL6, IL8, and VEGFA increased significantly in GPR4 infected cells at pH 5.9. (C) The supernatants of the cells were collected and the concentrations of IL6, IL8 and VEGFA were detected by ELISA. (TIF) [file pone.0152789.s001.tif]

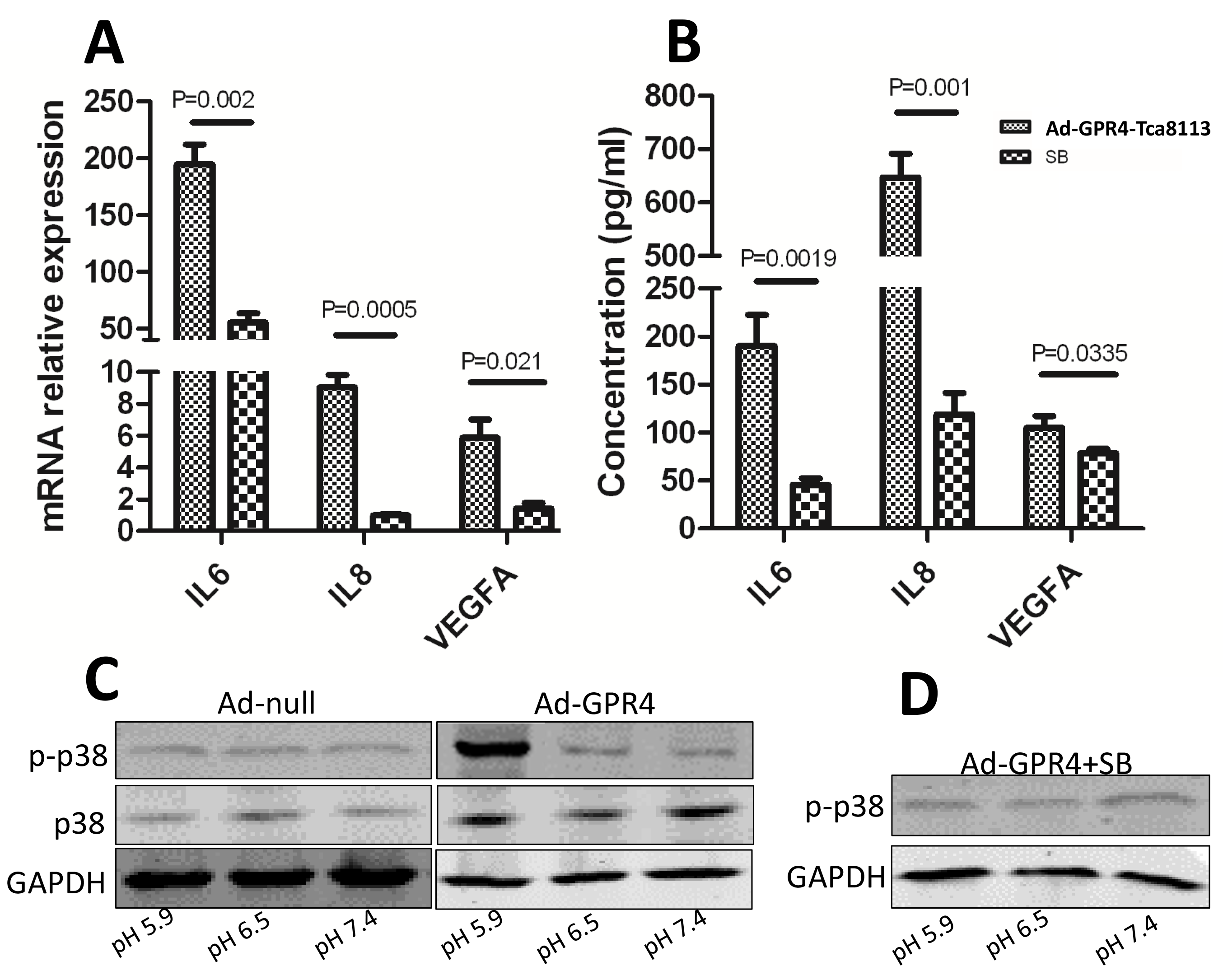

Supplement: S2 Fig — (A) In Ad-GPR4-Tca8113 cells, SB203580 reduced the expression of IL6, IL8 and VEGFA at pH 5.9. (B) SB203580 reduced secretion of IL6, IL8 and VEGFA. (C) GPR4 increased p38 phosphorylation at pH5.9. (D) SB203580 inhibit phosphorylation of p38 in GPR4 infected cells. (TIF) [file pone.0152789.s002.tif]

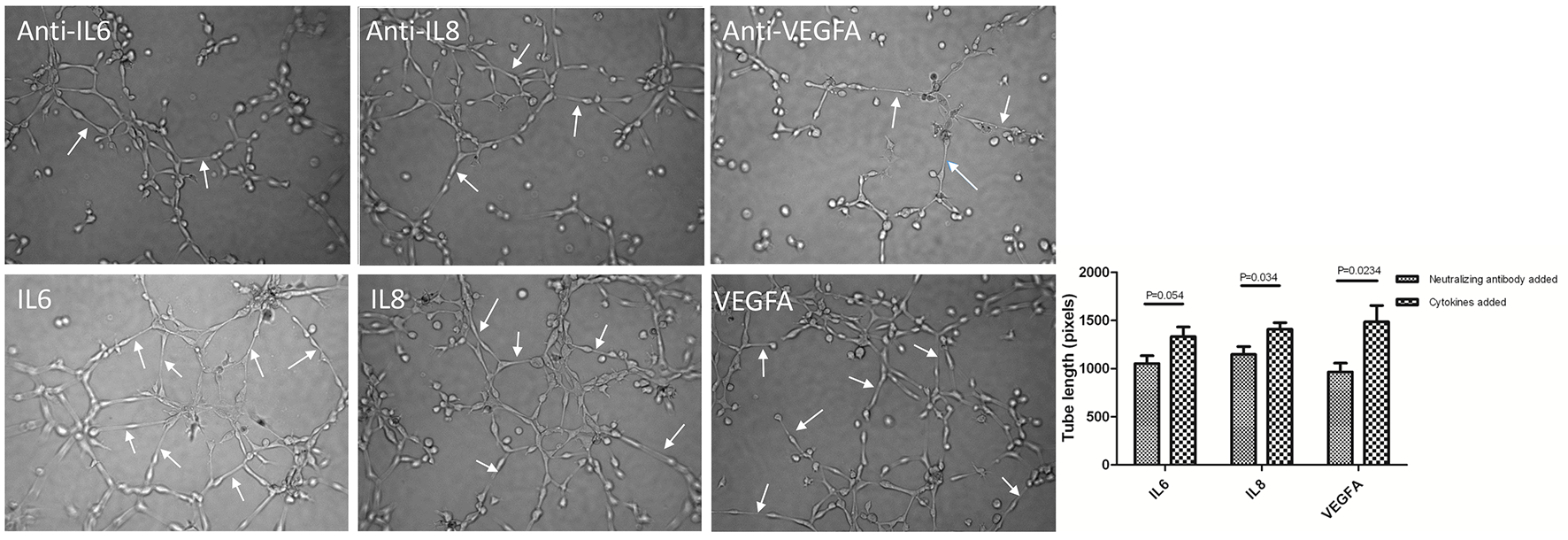

Supplement: S3 Fig — (TIF) [file pone.0152789.s003.tif]

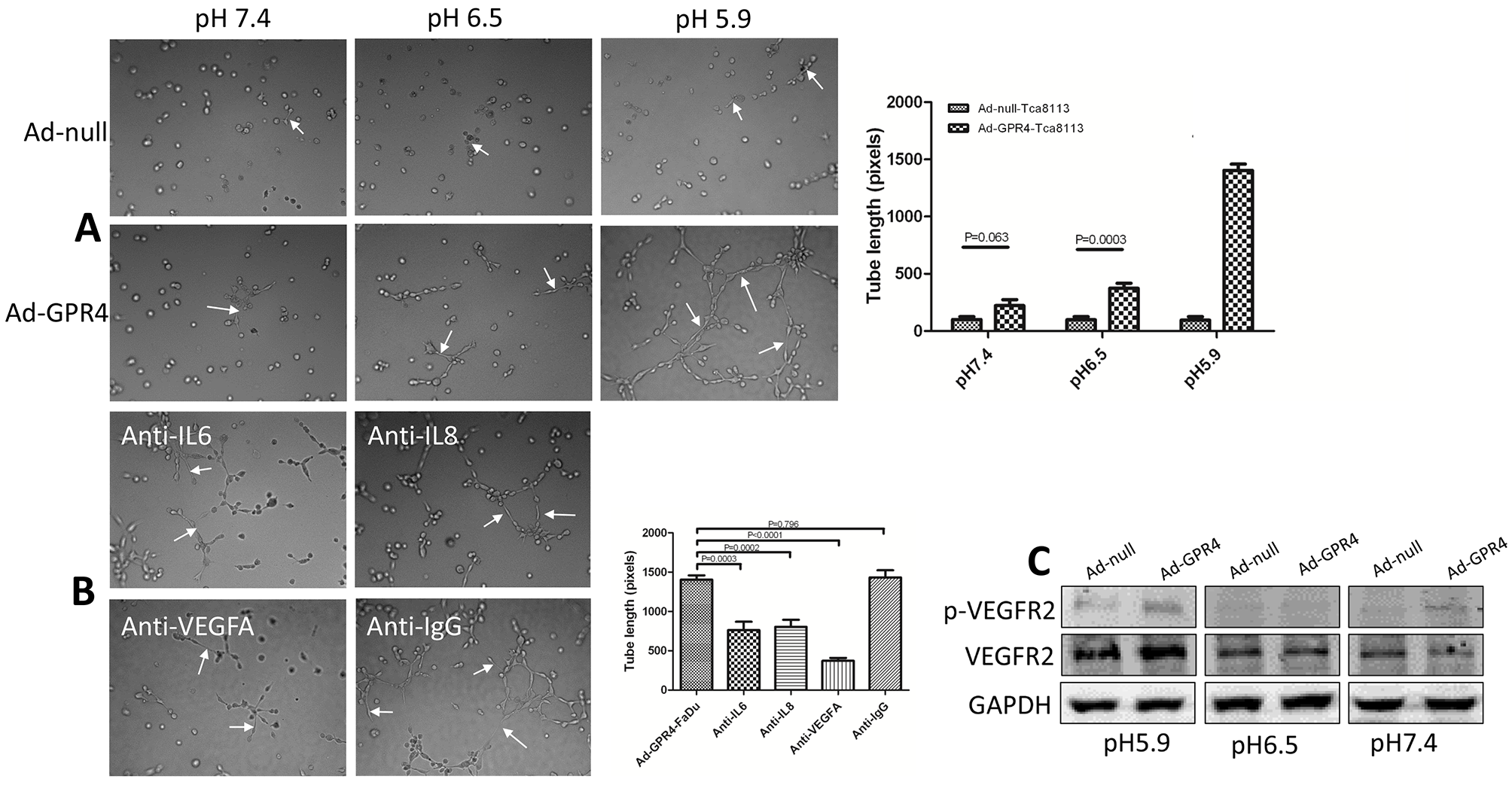

Supplement: S4 Fig — (A) The tube length (arrows) of HMEC-1 cells was increased in CM derived from Ad-GPR4-Tca8113 cells compared with Ad-null- Tca8113 cells at pH 5.9. (B) The neutralizing antibodies of IL6, IL8 and VEGFA inhibited tube formation in HMEC-1 cells. Isotype IgG antibody was used as a control in neutralizing antibody test. (TIF) [file pone.0152789.s004.tif]

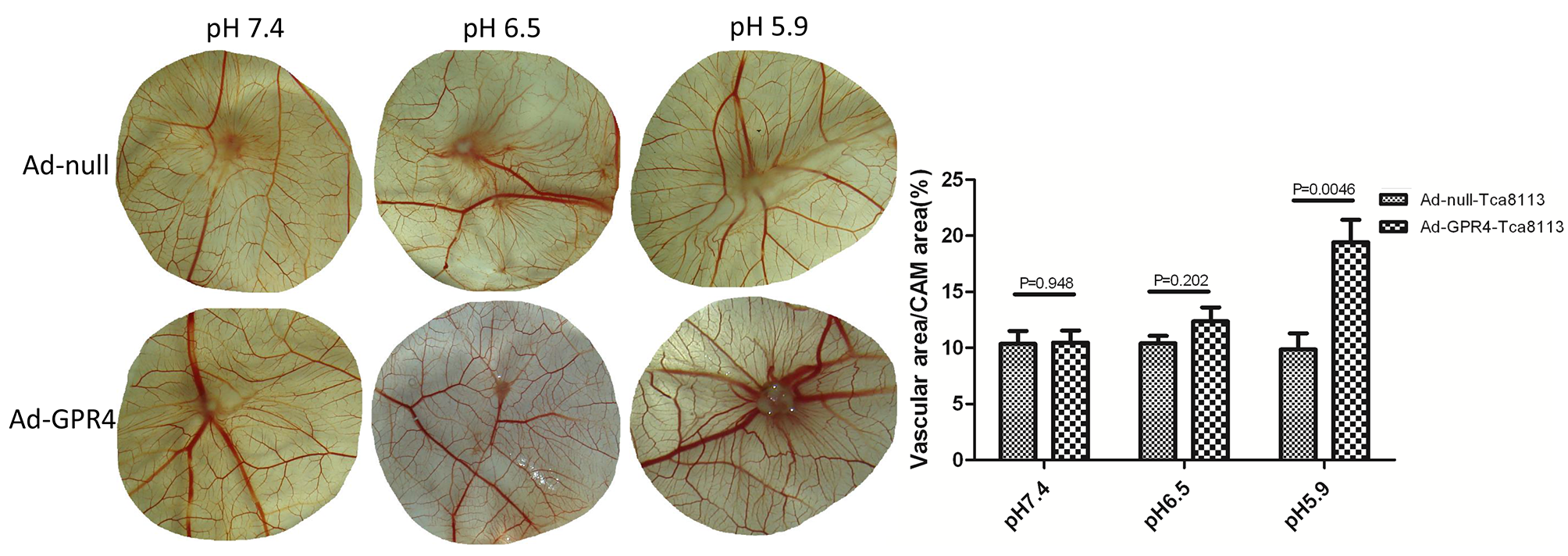

Supplement: S5 Fig — (TIF) [file pone.0152789.s005.tif]
